# Concomitant Persistent Left Superior Vena Cava and Horseshoe Kidney

**DOI:** 10.1155/2015/178310

**Published:** 2015-01-13

**Authors:** Faraz Jaffer, Vijay Chandiramani

**Affiliations:** University of Arizona, South Campus, Tucson, AZ 85715, USA

## Abstract

Persistent left superior vena cava (PLSVC) and horseshoe kidney (HSK) are common congenital abnormalities; however presence of both in the same person is extremely rare. A patient with hepatitis C cirrhosis awaiting transplant presented with worsening liver dysfunction, diagnosed with acute renal failure secondary to hepatorenal syndrome, and required X-ray fluoroscopy guided tunneled venous catheter placement for hemodialysis. Review of imaging studies demonstrated coexistence of PLSVC and HSK. PLSVC in adulthood is usually incidental with the most common drainage pattern being without physiologic dysfunction. Isolated horseshoe kidney is still the most common of renal fusion anomalies; however etiology of coexistent PLSVC remains unknown.

## 1. Introduction

Persistent left superior vena cava (PLSVC) and horseshoe kidney (HSK) are common congenital abnormalities. The prevalence of PLSVC and HSK in the general population is 0.3–0.5% and 0.1–0.3%, respectively. Both PLSVC and HSK are known to be physiologically insignificant in most patients. The presence of both PLSVC and horseshoe kidney arising in the same person is extremely rare with concurrent prevalence estimates unknown.

## 2. Case Description

A 58-year-old female with medical history significant for liver cirrhosis secondary to hepatitis C infection, chronic obstructive pulmonary disease, diabetes mellitus, and hypertension presented to the hospital for increasing abdominal distension, weakness, and confusion. She was first diagnosed with chronic nonhepatitis B hepatitis in 1983, later revised to hepatitis C cirrhosis in 1992, and underwent pegylated interferon plus ribavirin treatment for three months in 2002; however, she ceased treatment due to adverse effects leading to noncompliance. Since 2011, the patient has been undergoing evaluation for liver transplantation on outpatient basis and no prior hospitalizations were reported due to complications from liver disease.

**Figure 1:**
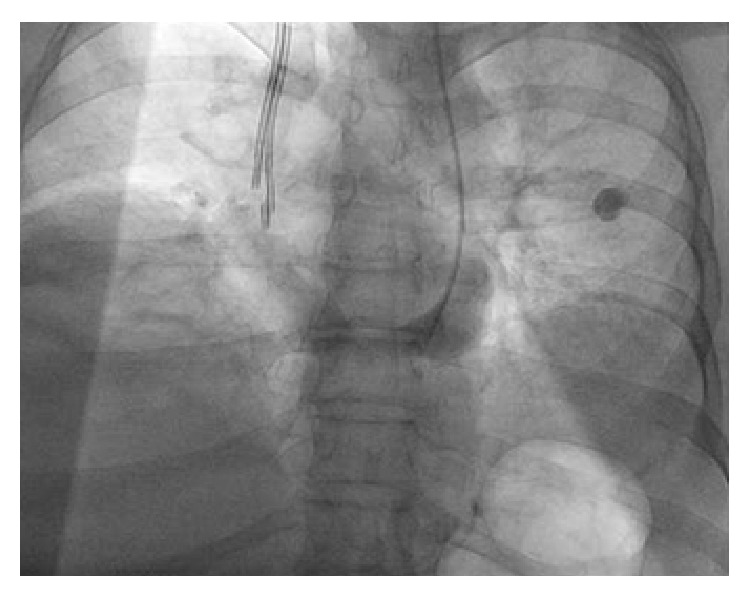
Persistence of left superior vena cava during X-ray fluoroscopic placement of tunnel dialysis catheter.

**Figure 2:**
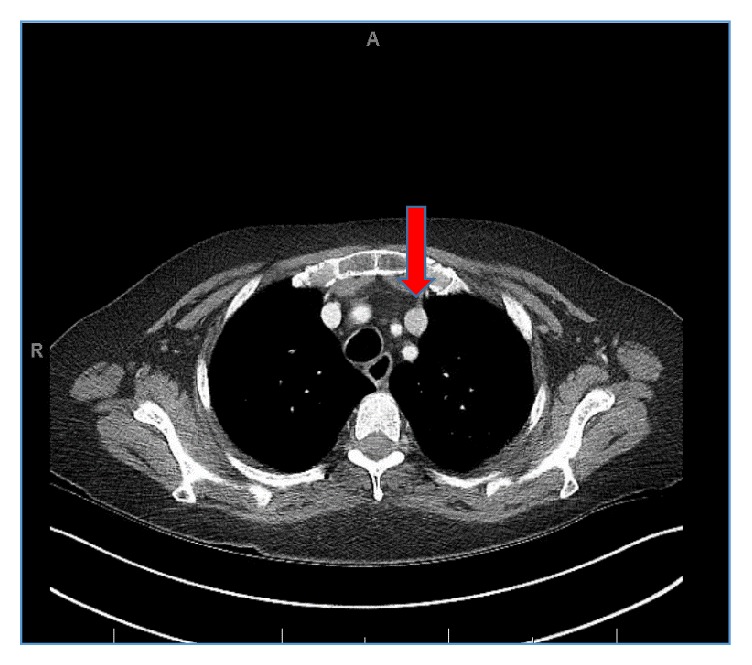
CT thorax visualizing persistent left superior vena cava.

At presentation, patient's encephalopathic and physical examination was remarkable for abdominal distension with tenderness. Laboratory findings demonstrated serum sodium of 118 mMol/L, potassium of 6.4 mMol/L, chloride of 114 mMol/L, blood urea nitrogen of 57 mg/dL, and creatinine of 4.8 mg/dL (baseline: 0.8 mg/dL). The patient was found to be oliguric, urine microscopy demonstrated “muddy brown casts” consistent with acute tubular necrosis, and after extensive workup this was determined as secondary to hepatorenal physiology. Patient's renal status continued to deteriorate to eventual anuria, hyperkalemia refractory to medical management, and encephalopathy, for which a vascular catheter was fitted into the right internal jugular vein under ultrasound guidance without difficulty and care, and the patient was transferred to the intensive care unit for continuous venous-venous hemofiltration (CVVH) therapy. The patient received a 5-day total of CVVH and, on hospital day 7, with creatinine clearance of 15.32 mL/min, the patient shuttled to catheterization lab, where 15 mL of Isovue-300 contrast was used for safe placement of a right-sided tunneled venous catheter for scheduled triweekly hemodialysis sessions. Contrast enhanced X-ray fluoroscopy demonstrated persistent left superior vena cava (Figure 1). Upon careful review of current and past imaging studies, a contrast enhanced computed tomography (CT) of the thorax, abdomen, and pelvis and a magnetic resonance imaging (MRI) of abdomen which were conducted as part of outpatient workup for liver transplantation corroborated the existence of both PLSVC and HSK (Figure 2).

## 3. Discussion

**Figure 3:**
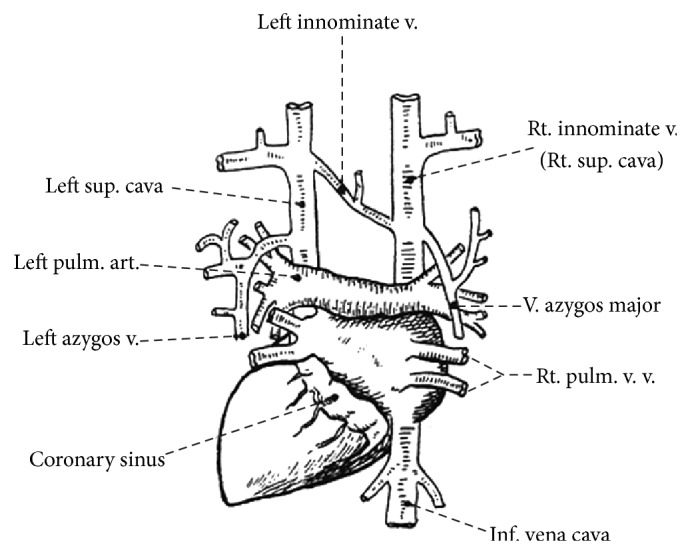
Diagram of the heart and great veins from behind, showing the arrangement in a case of persistence of the left superior cava. Credit: Sharpey-Schafer et al. [2].

Genesis of PLSVC is due to failed regression of left anterior cardinal with or without persistent left innominate vein. Our patient presentation is consistent with the most common type of PLSVC which demonstrates both left and right SVCs draining into the right atrium via coronary sinus without physiological consequence (Figure 3). The less common presenting drain pattern, seen in 10–20% of patients with PLSVC, results in communication with left atrium leading to right-to-left shunt that can create paradoxical emboli leading to neurologic, mesenteric, and/or peripheral sequelae. Most are incidental findings on chest imaging with widened mediastinum on plain chest film, and computed tomography of chest provides a more defined image. In our case, prior CT imaging documented left superior vena cava and however was not readily available for review when patient underwent catheter placement. Intraoperative venography can be utilized to map venous abnormality. Cognizant of the patient's impaired creatinine clearance, we scheduled the procedure to precede patient's scheduled dialysis session and elected to conservatively use contrast in our fluoroscopy to ensure safe and appropriate placement of catheter and thus minimizing complications such as fatal perforation of superior vena cava reported in the setting of unrecognized stenosis due to prior trauma from intravenous catheter [1]. Although the most common types of PLSVC have not been known to be symptomatic, intravenous placement and their implications in these rare anomalies are yet to be demonstrated.

Concomitant presence of horseshoe kidney highlights the rarity of finding two common congenital abnormalities in the same person. Isolated horseshoe kidney is the most common of renal fusion anomalies and patients clinically present with acute renal failure due to anatomic complications of ureteropelvic junction obstructions, nephrolithiasis, and less commonly malignancies, which can all be treated successfully through endourological intervention. A retrospective analysis of patients with known history of HSK compared with general population demonstrated significantly increased prevalence of PLSVC; however, patients were asymptomatic and no reasons for their coexistence were identified. Even though there are no complications reported due to concurrent HSK with PLSVC, the etiology of their coexistence is unknown and thus warrants further investigation. In addition, it would be of use to investigate whether prevalence of horseshoe kidney retards recovery from acute kidney injury compared to general population.

Interventionists frequently utilize the left internal jugular as site of access for tunnel catheter placement; thus, identification and knowledge of anomalous vascular anatomy are vital to safe placement of tunneled central venous dialysis catheter.
